# Integrated vector management (IVM): from concept to practice

**DOI:** 10.1186/1475-2875-9-S2-I12

**Published:** 2010-10-20

**Authors:** Henk van den Berg, Willem Takken

**Affiliations:** 1 Laboratory of Entomology, Wageningen University,The Netherlands

## 

The effectiveness of vector control is commonly limited by several factors: Sub-optimal targeting and lack of adaptation of methods, missed opportunities for integrating diseases, other sectors and communities are insufficiently aware of their role in increasing disease risk, and insecticide resistance. IVM is a strategy to tackle these problems. Under the global IVM initiative, a global action plan on IVM has been developed by a group of stakeholders, facilitated by WHO. Principles of IVM are: Decision making based on local and contemporary conditions, adopting a multi-disease approach by considering all prevalent diseases, taking into account all relevant vector control methods, and involving other sectors and communities.

A process of decision making that incorporates these principles is proposed. The first three steps, epidemiological assessment, stratification, and vector assessment, depend on specialised expertise usually available only at central level. The next three steps, system analysis, selection of options and needs assessment, are best conducted at decentralised levels. Hence, decentralization is vital to IVM. A descriptive example of an IVM strategy, with pilot experience, is presented.

But IVM is more than field implementation. It requires new organizational structures, new roles and responsibilities, both within the health sector, and in partnership with other sectors. IVM promotes the 'embedding' of vector control within decentralized health systems. Potential benefits are: increased motivation and status of district staff, extended reach of services, synergies and cost savings in services, and increased recognition of vector control. Through partnerships, other sectors can address risks in their own sphere of influence. A National policy and governing body on IVM will be essential to establish inter-sector collaboration at central and decentralized levels. In addition, communities should take control of, and assume responsibility for, risk factors within the peri-domestic sphere. By emphasizing capacity building on analysis and decision making, the conditions for empowerment are inherent to the IVM approach. In the transformation of the existing systems to IVM, Health sector funds could become available if synergies and cost-savings become known. Other sectors could contribute by internalizing disease mitigating procedures in their programmes.

